# Intermittent-Aware Design Exploration of Systolic Array Using Various Non-Volatile Memory: A Comparative Study

**DOI:** 10.3390/mi15030343

**Published:** 2024-02-29

**Authors:** Nedasadat Taheri, Sepehr Tabrizchi, Arman Roohi

**Affiliations:** School of Computing, University of Nebraska-Lincoln, Lincoln, NE 68588, USA; stabrizchi2@huskers.unl.edu

**Keywords:** intermittent computing, systolic array, non-volatile memory, accelerator

## Abstract

This paper conducts a comprehensive study on intermittent computing within IoT environments, emphasizing the interplay between different dataflows—row, weight, and output—and a variety of non-volatile memory technologies. We then delve into the architectural optimization of these systems using a spatial architecture, namely IDEA, with their processing elements efficiently arranged in a rhythmic pattern, providing enhanced performance in the presence of power failures. This exploration aims to highlight the diverse advantages and potential applications of each combination, offering a comparative perspective. In our findings, using IDEA for the row stationary dataflow with AlexNet on the CIFAR10 dataset, we observe a power efficiency gain of 2.7% and an average reduction of 21% in the required cycles. This study elucidates the potential of different architectural choices in enhancing energy efficiency and performance in IoT systems.

## 1. Introduction

As we enter a new era of computation, characterized by an exponential increase in the complexity of tasks being performed, the field of deep learning has been experiencing monumental advancements. These advancements have been fueled by the development of novel hardware, namely deep learning accelerators, which are specifically designed to facilitate complex computation and data processing tasks associated with deep learning algorithms. Deep learning accelerators, an embodiment of application-specific integrated circuits (ASICs), exhibit an exceptional capacity for high-speed, large-scale computation. These dedicated hardware units comprise an array of processing elements (PEs) designed to optimize the computational efficiency of deep learning algorithms. The intricate design of these accelerators allows for the simultaneous execution of multiple operations, dramatically reducing computational latency and providing unprecedented computational power. However, the conventional design strategies employed in deep learning accelerators often overlook energy efficiency, a critical parameter in the current scenario of constrained energy resources and green computing initiatives. The PEs, the atomic units of these accelerators, play a fundamental role in determining overall performance and energy consumption. They perform computations at an extraordinary speed but consume considerable energy in the process. A careful examination and optimization of these PEs could, therefore, substantially improve both the energy efficiency and performance of deep learning accelerators [1,2]. On the other hand, energy harvesting systems provide a sustainable solution to the energy constraints of these accelerators. They have the potential to provide continuous power harvested from ambient sources, thereby extending the operational life and enhancing the overall efficiency of deep learning accelerators. Recent developments in energy harvesting technologies have demonstrated promising advancements, yet there is still much room for improvement. In this context, the demand for an intermittent, energy-aware design scheme is increasingly becoming evident. To address this need, our study conducts a comparative analysis of different dataflows, row-stationary (RS), weight-stationary (WS), and output-stationary (OS), utilizing various non-volatile memory (NVM) technologies. We extend beyond the scope of traditional architectures by examining these combinations in the context of intermittent-aware design. This approach not only offers insight into the performance enhancements achievable through these diverse configurations, but also highlights the potential for improved energy efficiency. For instance, we considered a comprehensive approach, which can optimize both the performance and energy efficiency of deep learning accelerators by focusing on their PEs and integrating energy harvesting systems [3,4].

This paper introduces a detailed exploration of these dataflows and NVM technologies using IDEA architecture, which is implemented as part of our examination using different dataflows. We showed how different dataflow strategies and NVM choices can significantly impact energy efficiency and performance using intermittent-aware deep learning accelerators.

## 2. Background

At the heart of any ASIC machine learning accelerator lies the central principle of dataflow processing, a method with profound implications for data reuse. From a high-level perspective, dataflow processing comes in various forms, each with its inherent benefits and limitations. The static dataflow architecture is where the entire dataflow graph is compiled prior to execution. The advantages of such a scheme are evident in its increased data reuse and commendable power efficiency, underpinned by a reduced memory footprint. However, it poses challenges in accommodating dynamic alterations in model architectures, an inherent trade-off for its stellar power efficiency and lower memory requirements. On the contrary, dynamic dataflow architectures curate their dataflow graph during runtime. This dynamism fosters adaptability and is particularly appropriate for rapidly evolving machine learning models. The price paid for such flexibility manifests in lower data reuse, diminished power efficiency, and a more substantial memory footprint compared to its static counterpart [5]. Straddling these two extremes is the hybrid dataflow architecture, a system that blends static and dynamic elements. This union encourages flexibility while optimizing computational efficiency. However, the complexity of its implementation poses a noteworthy challenge. Moreover, several dataflow optimization techniques are commonly used in ASIC accelerators for deep learning, especially to optimize matrix multiplication and convolution operations, known as stationary dataflows. There are a variety of stationary dataflows, each keeping a specific type of data fixed (“stationary”) within the PEs to maximize data reuse. In the WS method, the neural network weights remain stationary. Although this technique allows for significant weight reuse, it potentially falls short when the reuse of input and output data is high. On the contrary, the input stationery technique emphasizes the reuse of input data, leading to potential inefficiencies when weight reuse is high. The OS (or Partial Sum Stationary) method keeps partial sums stationary, attempting to optimize both input and weight reuse. The trade-off comes with a higher requirement for local storage and potential inefficiencies when the same output is not used repeatedly. The RS (Column Stationary) technique, on the other hand, maintains a whole row (column) of data stationary. This approach aims to balance input, weight, and output reuse, but it introduces higher design and control complexity and necessitates additional local storage. The choice of the appropriate dataflow architecture and stationary dataflow type in an ASIC accelerator is challenging and hinges on a multitude of factors, including the complexity and specifics of machine learning workloads, adaptability requirements, power constraints, and performance goals [6,7,8,9].

### 2.1. Energy Harvested System

#### 2.1.1. Ambient Energy Sources

**Figure 1:**
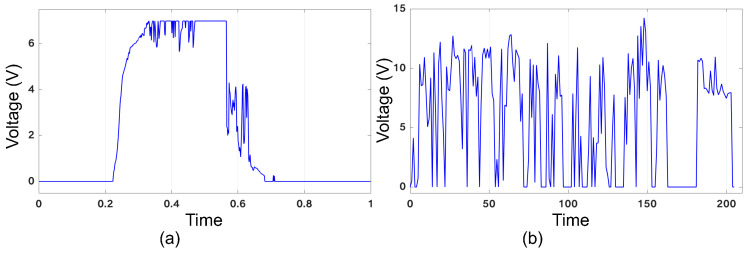
Voltage traces of (**a**) solar and (**b**) wind sources [16].

Ambient energy sources, such as solar and wind power, are vital in pursuing sustainable and renewable energy solutions. These sources harness energy from the environment and convert it into usable forms of power. Solar energy, derived from sunlight, can be captured through photovoltaic panels, while wind energy is harnessed by wind turbines. Both solar and wind power offer significant advantages, including abundant availability, reduced carbon emissions, and long-term cost savings [10,11]. Figure 1 illustrates the voltage traces from a building’s solar and wind energy systems throughout the day, revealing unique patterns. These patterns provide valuable information about how these ambient energy sources generate and consume energy. On the other hand, RFID (Radio Frequency Identification) technology uses a unique power source that distinguishes it from other electronic devices [12]. Unlike traditional devices that rely on internal batteries or wired connections, RFID systems draw power wirelessly from the reader or interrogator. This remarkable feature is enabled by electromagnetic induction. When an RFID reader emits a radio frequency signal, it creates an electromagnetic field that induces a small current in the antenna of the RFID tag. This induced current serves as the power source for the tag, allowing it to communicate and transmit data back to the reader. This innovative power supply method eliminates the need for bulky batteries or constant maintenance, making RFID an efficient and versatile solution for various applications, such as inventory management, access control, and supply chain optimization. Although high-energy bursts are beneficial, the intermittent nature of these sources can disrupt the operation, causing data loss or glitches and leading to unpredictable results [13,14,15].

Our research has focused on using the RFID source to design a specialized architecture. On the basis of the observed behaviors of this source, our proposed architecture considers the intermittency and variability of this energy source. By carefully designing our system to adapt to fluctuations in voltage levels and harness the available energy efficiently, we aim to optimize energy utilization and minimize dependency on non-renewable resources.

#### 2.1.2. Intermittent Computing

Energy-harvesting devices utilize intermittent computing, a technique characterized by short periods of program execution punctuated by power failures. Despite the inherent challenges associated with intermittent computing, numerous studies have concentrated on implementing techniques and systems that harness this form of computing across various levels, including hardware, software, and circuitry. Certain challenges must be taken into consideration during design. Resources are finite; thus, the less energy a device requires for computation, the more tasks it can execute. The execution time of each task can vary significantly, and on occasion, intensive tasks may be interrupted by power failures. This can lead to the expiration of data or computation before the task is completed. Developers of energy-harvesting devices require intuitive, valuable language and software support; they need the ability to interact with their sensors despite intermittent power supplies [17]. Moreover, they require comprehensive knowledge of both hardware and energy harvesting behaviors. Additionally, some tasks are atomic and should only begin when sufficient power is available. This requires effective task scheduling and power management.

Software-level techniques for intermittent computing involve the use of checkpoints and task-based programming models [18]. Checkpointing enables the preservation of volatile states in non-volatile memory (NVM), allowing computation to resume after a power failure. Meanwhile, task-based systems break programs into short tasks and resume execution of the last completed task. Task-based systems often employ various memory management techniques, such as privatization and variable copying. Additionally, there are hardware modifications and approaches based on speculation and watchdog timers to optimize data storage and restore tasks during power failures. Programming languages such as Chain and Mayfly simplify intermittent computing by segmenting programs into tasks and managing time and memory consistency. Compiler-based approaches analyze program sections and determine the minimum number of checkpoints needed to prevent incompatibilities. These software-level techniques and languages offer strategies to overcome the challenges of intermittent computing and facilitate efficient operation in energy harvesting systems.

Architecture-level techniques for intermittent computing offer various solutions to overcome the challenges of power failures and optimize energy efficiency [19,20]. Approximate computing techniques are used to reduce the accuracy of computations for improved performance and efficiency. Checkpointing schemes based on FRAM-based unified memory and SRAM-NVM hybrid caches minimize overhead and enhance data storage and retrieval. Machine learning approaches dynamically adjust the microarchitecture and resource allocation based on power supply conditions. Hardware solutions such as Clank track memory access to detect write-after-read sequences, while the Cascaded Hierarchical Remanence Timekeeper (CHRT) provides resilient timekeeping during power failures. Hardware/software co-design approaches allow for dynamic reconfiguration of energy buffers and task-based execution. Non-volatile processors (NVPs) integrate NVMs for an instant on/off execution and reduced leakage power. FPGA-based designs and TFETs are explored for efficient intermittent computing systems. To push the boundaries of energy harvesting devices and intermittent computing, a co-design approach is vital, entailing the development of novel hardware and software strategies that optimize AI applications on these intermittent computing systems. Exploring combinations of techniques, such as integrating NVM into deep learning approaches, can further enhance the capabilities of these systems. This paper proposes an innovative approach by combining the aforementioned techniques with integrating NVMs. Taking advantage of the unique characteristics and capabilities of NVM, we enhance the performance and efficiency of intermittent computing systems. By exploring intermittent-aware accelerators comprehensively, this paper contributes to the advancement of the field and provides valuable insight for future energy-efficient computing.

### 2.2. Emerging Non-Volatile Memory

By providing persistent storage, NVMs play an important role in battery-less systems. Unlike volatile memories, NVMs retain stored information without a continuous power supply. This characteristic makes them well-suited for applications requiring long-term data storage and reliable operation. Several types of NVMs have been developed, including Resistive Random Access Memory (ReRAM), Ferroelectric RAM (FeRAM), non-volatile random access memory (nvSRAM), Spin-transfer Torque Magnetic Random Access Memory (STT-MRAM), and Spin-Orbit Torque Magnetic Random Access Memory (SOT-MRAM). Each of these offers unique advantages in terms of performance, scalability, endurance, and power efficiency. We evaluate our proposed architecture with all the mentioned technologies based on Table 1.

ReRAM provides high density, low power consumption, and fast switching speed, making it an attractive alternative to traditional memory technologies. ReRAM works by reversibly changing the resistance of dielectric solid-state material between high and low states using the phenomenon of resistive switching. This unique behavior enables ReRAM to store and retrieve data effectively. Furthermore, ReRAM’s compatibility with existing CMOS fabrication processes makes it a promising candidate for next-generation memory devices. ReRAM has been shown to have potential applications in neuromorphic computing [22].

FeRAM utilizes the unique properties of ferroelectric materials, which exhibit spontaneous polarization that can be reversed by an external electric field. As a result of this characteristic, FeRAM is able to store data in a non-volatile manner. Based on Table 1, FeRAM consumes less power in comparison to nvSRAM and ReRAM and is extremely durable. The read and write speed is lower compared to all other examined memories. These attributes make FeRAM an attractive candidate for unreal-time applications, such as intermittent computing [23].

nvSRAM is an innovative type of memory that combines the benefits of SRAM and non-volatility. Unlike traditional SRAM, which loses data when power is cut, nvSRAM utilizes non-volatile storage elements, such as ferroelectric or magnetic materials, to retain data even without power. This unique property of nvSRAM makes it highly desirable for applications that require both fast and reliable data access, as well as data persistence during power outages. nvSRAM offers the advantage of instant data availability upon power-up, eliminating the need for time-consuming data restoration processes. A major goal of nvSRAM is to have a minimal impact on SRAM’s fundamental structure. While many nvSRAMs have been presented, including 4T2R, 7T1R, and 8T2R, herein, 7T1R [21] is leveraged.

STT-MRAM operates based on the phenomenon of spin transfer torque (STT), where a spin-polarized current is used to manipulate the magnetic orientation of a storage element. This offers advantages such as high endurance, fast read and write speeds, and low power consumption. Its compatibility with standard CMOS processes makes it an attractive option for next-generation memory architectures [24].

SOT-MRAM has been identified as one of the best NVM technologies based on Table 1, which shows great promise in recent research. SOT-MRAM utilizes the spin–orbit interaction to manipulate the magnetization direction of the storage element. By applying an electric current through a heavy metal layer, the spin–orbit torque can induce a change in the magnetic state, allowing for non-volatile data storage. SOT-MRAM offers several additional advantages, including fast switching speed, low power consumption, and scalability. Ongoing research is focused on improving the performance and reliability of SOT-MRAM, as well as exploring its integration with existing semiconductor technologies [24].

### 2.3. Recent Studies

Recent advancements in intermittent computing and NVM technologies have paved the way for significant improvements in energy efficiency and system reliability, particularly in the context of energy-constrained devices and systems. The integration of these technologies plays a crucial role in the development of computing systems capable of operating under fluctuating power conditions while minimizing energy consumption and data loss. In exploring this field further, various research efforts have focused on optimizing data movement and computation in DNN accelerators, employing strategies such as RS, WS, and OS to improve performance and efficiency. RS techniques aim to minimize the movement of input feature map rows by keeping them stationary within the processing elements. This strategy is beneficial in reducing the energy consumption associated with data movement. Significant contributions in this area include the development of resource-efficient convolutional networks and the proposition of low-complex mapping algorithms for spatial DNN accelerators [25,26,27,28,29,30]. WS methods focus on keeping the weights within the processing elements stationary, optimizing the reuse of weights across different operations to minimize data transfers [31]. This approach is highlighted in different studies [32,33,34], which explore dynamic resource allocation and the design of accelerators for efficient matrix multiplication. All these contributions demonstrate the versatility and potential of WS in various applications [35,36]. On the other hand, OS strategies ensure that the output data remain stationary within the processing elements, thereby optimizing the computation of outputs and reducing the need for data movement. This concept is elaborated in different studies [37,38,39] which discuss design and optimization strategies for systolic array-based and FPGA-based DNN accelerators. Other studies, such as [40,41], furthermore, explore the benefits of OS in enhancing computational efficiency. Each of these strategies has distinct advantages in the design and optimization of DNN accelerators, addressing specific challenges related to data movement and computational efficiency. The next section will delve into the “Proposed Intermittent-Aware Design”, comparing these strategies against various non-volatile technologies to highlight their contributions to energy efficiency and system reliability in energy-constrained computing environments.

## 3. Proposed Intermittent-Aware Design Exploration

**Figure 2:**
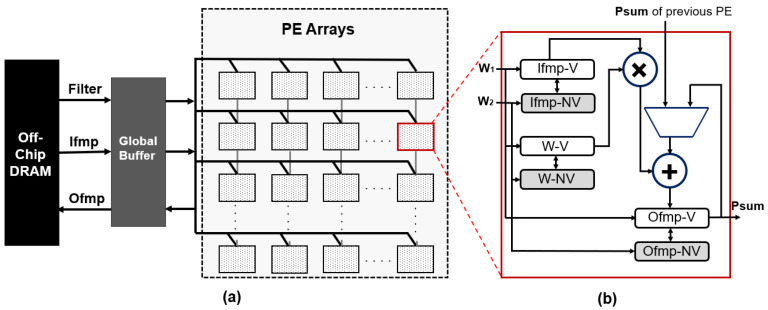
(**a**) The IDEA architecture and (**b**) processing element.

In this section, we aim to compare three dataflow strategies (RS, WS, and OS) alongside various non-volatile technologies. To facilitate this comparison, we will utilize a simulated architecture known as IDEA, which serves as the backbone for our analysis and experimentation. This exploration is grounded in the spatial organization of PEs configured in a 32×32 matrix, akin to the configurable systolic arrays found in contemporary accelerator generators. The configuration allows for dynamic dataflow alterations, supporting both output-stationary and weight-stationary dataflows, reminiscent of the flexibility seen in state-of-the-art accelerator templates. Figure 2a depicts the top-level structure of the architecture used for implementation. The system’s structure encompasses a spatially organized array of 1024 PEs assembled in a square matrix of 32×32 for our architecture [42]. This is complemented by an 864-kB Global Buffer (GLB). Each PE’s ability to interface with adjacent PEs or the GLB via the NoC system is reflective of the intricate interconnect networks that enhance data transfer efficiencies in advanced DL accelerators. For computational data transfer purposes, each PE can interface with its vertically adjacent PEs or interact with the GLB, facilitated by a network-on-chip (NoC) system. Modified PE architecture comprises three types of registers: input feature map (ifmap) buffer, Weight (W)/Filter buffer, and partial sum (psum) buffer. Each buffer’s capacity to interface with non-volatile backup memory options for the data retention during power loss is a testament to the robustness of memory hierarchies that are crucial in minimizing data loss and optimizing energy use, mirroring the accumulator and scratchpad memory parameters configurable in modular accelerator designs. Each buffer can have a non-volatile backup memory, ensuring data retention even during power loss. In this section, we explore how integrating different NVM technologies with these buffers will impact the resilience and energy efficiency of the IDEA architecture in various dataflow scenarios. Figure 2b illustrates the structure of the PE, where the buffers suffixed with “V/NV” represent volatile/non-volatile memories. In intermittent computing environments, incorporating NVM technologies is crucial for minimizing data loss and maximizing energy efficiency. To facilitate data access and storage in NVMs across all PEs, two universal write signals ($w_{1}/w_{2}$) are employed for writing from NV/V to V/NV memories. These control lines, interconnected with the power management unit, underscore the architectural emphasis on power-aware control mechanisms, which is a shared concern in designing accelerators that support a wide array of power and performance trade-offs.

**Figure 3:**
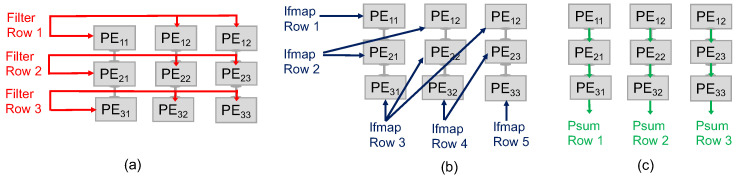
The RS dataflow in a 3×3 PE array for processing a 2D convolution. (**a**) Filter rows are consistently shared between rows of PEs. (**b**) The ifmap rows are applied diagonally to the PEs. Thus, diagonally adjacent PEs receive the same ifmap value. (**c**) Finally, the psums are propagated and accumulated vertically across PEs.

The IDEA architecture is inspired by optimized dataflows and NVM checkpointing. Figure 3 shows the dataflow offered by our architecture.

**Figure 4:**
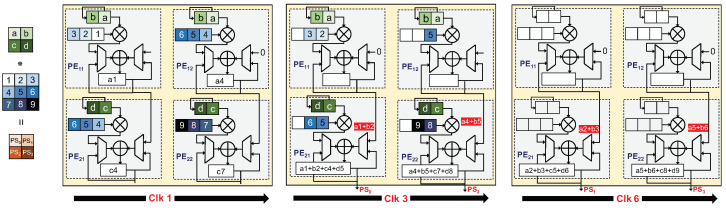
Example of RS dataflow in a 2×2 PE set for processing a 2D convolution.

    We considered three different dataflows, including RS, WS, and OS. The RS dataflow allocates the computational tasks related to any given Convolutional Neural Network (CNN) configuration across the PE array. This dataflow method simultaneously reduces the movement of all data categories, including ifmaps, filters, and psums/ofmaps. In this dataflow approach, each primitive performs operations on a single row of filter weights and ifmap values, thereby producing a single row of psums. These psums are subsequently combined to generate the output feature map (ofmap) values. By assigning each primitive to a specific PE for execution, the computation of each row pair remains stationary within that PE. We organize a group of PEs, depicted as a PE Set in Figure 3b, to conduct a 2D convolution. This arrangement exploits data reuse between primitives and the accumulation of psums, thus avoiding the need to access data from the GLB and off-chip DRAM. According to the proposed dataflow architecture, different operational characteristics are applied to different directional orientations. Specifically, the process of filtering rows will be conducted horizontally, applying the ifmap will occur diagonally, and accumulating the partial sums will be implemented vertically. This model integrates the use of two distinct shift registers. For the ifmap, a linear shift register is employed, which features a shift-enable functionality based on the stride size. This allows for the systematic and sequential processing of the ifmap data, facilitating efficient resource allocation and control flow. On the other hand, the filter utilizes a Linear Feedback Shift Register (LFSR), which instigates a shift in the register at each clock cycle. Moreover, the architecture incorporates two multiplexers, which are instrumental in directing the input for addition operations. Based on the enable signals of the multiplexer, the adder will add the output of MAC operation to the stored psum or the stored psum with the output of the adjacent PE. In the context of matrix computations, when employing a 2×2 matrix as a filter and a 3×3 matrix as an input feature map, every PE performs the MAC operation, as illustrated in Figure 4.

Furthermore, the non-volatile register keeps its data even when there is a reset. The volatile memory temporarily stores information until the power level reaches the backup threshold. When that happens, the data are saved in the non-volatile register until the power level increases again. This allows the accelerator to quickly resume computations using the saved data from the non-volatile register.

**Figure 5:**
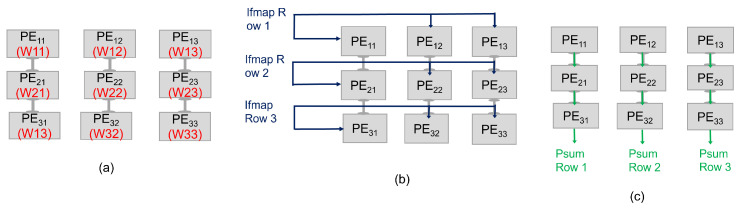
The WS dataflow in a 3×3 PE array for processing a 2D convolution. (**a**) Weights are stationary inside each PE. (**b**) The ifmap rows are consistently shared between rows of PEs. (**c**) Finally, the psums are propagated and accumulated vertically across PEs.

**Figure 6:**
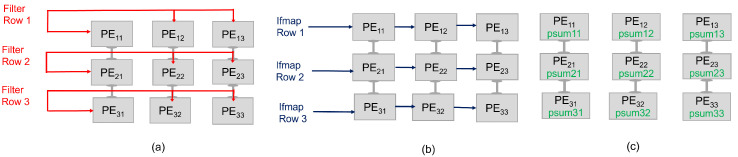
The OS dataflow in a 3×3 PE array for processing a 2D convolution. (**a**) Filter rows are consistently shared between rows of PEs. (**b**) The ifmap rows are propagated vertically across PEs. (**c**) Finally, the psums are stationary inside PEs.

We extend our exploration to include the WS and OS dataflows, depicted in Figure 5 and Figure 6, respectively. These dataflows are examined alongside the RS to provide a comprehensive view of how different dataflow strategies impact the performance and efficiency of the IDEA architecture when integrated with NVM technologies. In the WS, each PE is assigned a specific weight (or a set of weights), which remains constant throughout the computation process. The ifmap values are streamed across the PE array row by row, allowing for the simultaneous computation of the psum within each PE. This minimizes the need for data movement, particularly for the weights, which are this scheme’s most stationary data types. The effectiveness of the WS dataflow in conjunction with NVM technologies is particularly notable in scenarios requiring high-weight reuse. As the ifmap rows propagate through the array, each PE multiplies its stationary weight by the incoming ifmap value, accumulating the result into the psum. Once all the necessary ifmaps have been processed, each PE contains a complete psum for its respective output feature map row. The psums are then passed down the array or aggregated within the PEs to produce the final ofmap values. The WS dataflow is particularly effective in reducing memory bandwidth requirements and power consumption, as it avoids the frequent loading and unloading of weights, making it an efficient choice for hardware implementations of CNNs. On the other hand, in OS, each PE is dedicated to computing a single psum element. The psum values remain stationary within their respective PEs until the entire computation for that specific ofmap element is completed. This dataflow is especially efficient when paired with NVM technologies, as it further reduces the data movement of psums. In this dataflow, filter rows are fed horizontally across each PE row, while the corresponding ifmap rows flow vertically down the columns of PEs. The PEs perform multiply-accumulate operations on the incoming filter and ifmap data, updating the psums stored within each PE. These psums are the intermediate values that eventually make up the final ofmap elements. The key advantage of the OS approach is that it minimizes the movement of psums, which can be data-intensive to shift around the PE array. By keeping the psums stationary and only moving the filter and ifmap values, the architecture can reduce the data bandwidth requirements and power consumption, leading to more efficient processing.

### Architecture Behaviour

**Figure 7:**
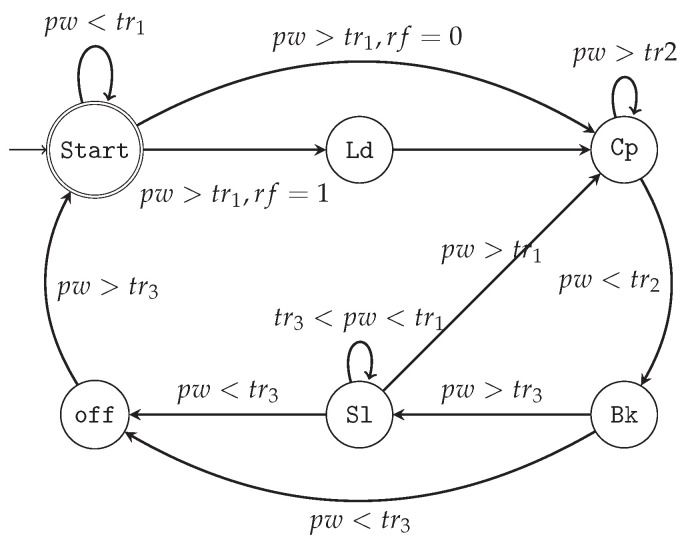
The proposed state machine considered in the IDEA architecture.

**Figure 8:**
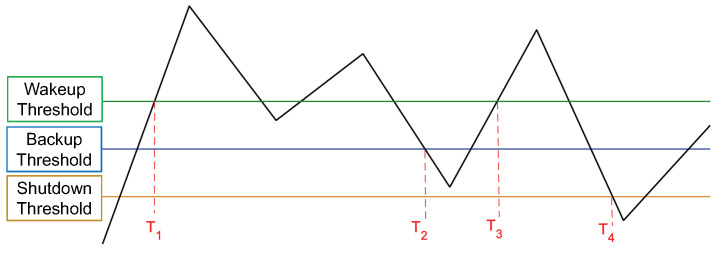
Defined thresholds for the evaluation; T_1_, where the system starts computing, T_2_, where the data is sent from volatile registers to non-volatile registers, T_3_, where computing starts again, and T_4_, where the system shuts down/goes to sleep.

The finite state machine of the system is presented in Figure 7, where there are three voltage thresholds, i.e., $tr_{1}$, $tr_{2}$, and $tr_{3}$, for wake-up, backup, and shutdown, respectively, which we considered as 2.5, 1.5, and 0.8 V. During system startup, the system initially enters the **Start** state. It remains in this state until the power supply voltage (pw) exceeds the first threshold value, denoted as $tr_{1}$. At this point, the system’s state can be changed to either Load (**Ld**) or Computation (**Cp**), based on the value of the register flag (*rf*). The *rf* is a one-bit register that indicates whether the backup process before the shutdown was successful or not. In **Ld**, the signal $w_{1}$ is set to ‘1’. Consequently, the values of all non-volatile registers are transferred into volatile registers, enabling the system to continue with the previous computation. If the backup process fails, all the volatile registers must be loaded from the off-chip memory. After loading the registers, the system transitions to the **Cp** state. The system remains in this state until the power supply voltage drops below the $tr_{2}$ threshold value. This threshold represents a critical voltage level, indicating the need to back up all the required memories. When this occurs, the system’s state is immediately changed to Backup (**Bk**). In this state, the $w_{2}$ is set to ‘1’, and the values of all volatile registers are copied into NVMs. If the system has sufficient power to complete the backup process, the state changes to Sleep (**Sl**), and the *rf* is set to ‘1’. However, if there is insufficient power to keep the system alive, the state transitions to the **off** state. **Sl** can be exited in two possible ways. If *pw* exceeds the threshold value $tr_{1}$, the system can return to normal operation. On the other hand, if the power supply voltage drops below the $tr_{3}$ threshold value, it indicates that the system is in the **off** state. Once enough energy is observed (i.e., the power supply voltage exceeds $tr_{3}$), the state machine restarts from the Start state. It is worth noting that in **Cp**, the value of the *rf* should be reset to ‘0’. The corresponding voltage thresholds and their impact on system behavior are illustrated in Figure 8.

## 4. Evaluation Platform

**Figure 9:**
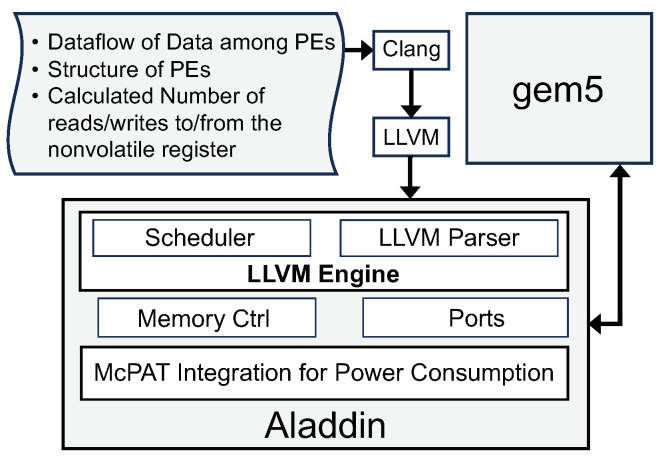
Proposed validation and evaluation framework.

This section provides an overview of the experimental setup used to investigate the performance of the proposed IDEA architecture. It encompasses particulars pertaining to the dataset harnessed, the assessment measures adopted, and the computational simulation environment instantiated for experimental evaluation. Furthermore, it describes the methodology used to compare different dataflows and NVM technologies in the context of intermittent-aware computing. To verify our approach’s functionality, using the IDEA architecture, we evaluate different dataflows and NVM technologies’ performance under similar conditions. A system-level in-house framework is developed, shown in Figure 9; we used the cycle-by-cycle dynamic scheduling for the cycle count from the scheduler component and also modified McPAT component code to consider the power consumption of Read and Write power usage of non-volatile registers. This modification allows us to accurately gauge the impact of different NVM technologies on the power efficiency of various dataflows. In our architecture, the Aladdin Simulator operates as a specialized, integrated framework for accurate modeling and performance simulation. The workflow begins with ingesting “C Code” as the primary input. An “LLVM Tracer” subsequently analyzes and traces this code, transforming the raw code into an optimized representation amenable to precise simulation. Post-transformation, the data are channeled into the “LLVM Engine”. During this step, the engine interacts with embedded memory and CPU models, facilitated by the gem5 simulation environment. This synergistic interaction enables the computation of two critical performance metrics: power consumption and cycle count. Through this intricate process, the Aladdin Simulator block provides comprehensive, multi-faceted insights into the efficiency and operational dynamics of the system under study.

### 4.1. Experimental Setup

In our experimental setup, we simulate the RS, WS, and OS dataflows, drawing inspiration from [25,31,37], to evaluate their performance in the context of convolution operations under intermittent power conditions. The IDEA architecture comprises a PE set designed to maximize locality and minimize unnecessary data movement, with the added dimension of examining how different NVM technologies affect these aspects. When exploring different dataflows, we adjust the PE set size and configuration to suit the specific requirements of each dataflow strategy, providing a comprehensive comparison of their effectiveness in various scenarios. Given that convolutional layers account for approximately 90% of the overall power consumption in neural networks, our utilization of the mentioned dataflow significantly improves efficiency. Our architecture comprises the mentioned PE set to maximize locality and minimize unnecessary data movement, effectively computing the output for the target dataset. The PE set size is determined based on the specific requirements of the deep CNN being executed. Factors such as the network’s layer sizes, parallelism considerations, and memory constraints may influence the determination of the PE set size. When this number surpasses our architecture’s capacity, we employ a partitioning approach, specifically tiling, to divide the computation into smaller subsets. On the other hand, when the computed number is less than the capacity, we engage the necessary number of PEs. The remaining PEs are then designated to assume the role of routers within the system.

### 4.2. Non-Volatility and Intermittency Behaviors

As previously explained, each PE is equipped with two registers (one volatile and one non-volatile). To mimic the function of non-volatile registers, we adopted the method proposed in the NORM architecture [20]. We implemented a signal that interconnects all components, excluding non-volatile registers. Upon activation of the reset signal, the volatile registers are purged, while the non-volatile registers remain untouched by the reset operation. Data are held in the volatile memory until the power level descends below the second threshold. At this juncture, the data are backed up and maintained in the NVMs until the power level ascends above the wake-up threshold. Following this, we validate whether the data have indeed been preserved in the non-volatile register. If confirmed, these stored data are then utilized for the following computations. We conducted six different variations in our study to examine various scenarios. In the first scenario, we utilized NVMs for all weights, ifmaps, and psums. The second scenario involved non-volatile registers for weights and psums, while the third scenario employed them for ifmaps and psums. The remaining scenarios exclusively employed NVMs for weights, ifmaps, and psums, respectively.

**Figure 10:**
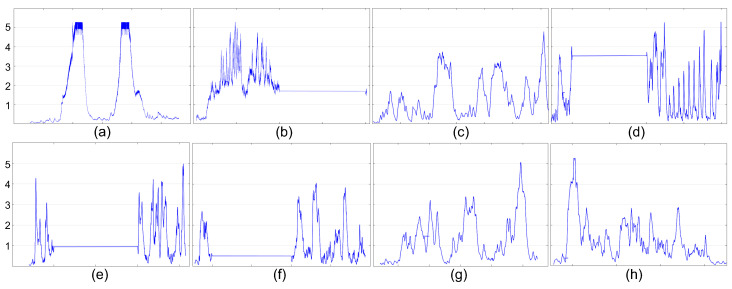
Different voltage traces of RFID sources, (**a**–**h**), utilized in the evaluation.

To incorporate the behavior of intermittency into our study, we adopted a methodological approach that involved simulating an intermittent power source characterized by a predetermined sequence of voltage levels that cyclically repeat. In order to accurately represent the behavior of intermittent power, we introduced a virtual energy source within our simulation framework, designed to mimic the functionality of a battery. This virtual energy source was responsible for accumulating energy during periods of power availability and deducting energy consumption during periods of power unavailability. Throughout the simulation process, we closely monitored several key parameters to ensure an accurate representation, including power/voltage levels, power availability, and the level of the virtual energy source. Monitoring was achieved by observing the behavior of the power source and virtual battery, as well as tracking the power consumption associated with various computational operations. Specifically, we assign a power consumption value of 5.03 pJ for each MAC operation [43]. To conduct our simulations, we utilized different voltage traces, as shown in Figure 10, regarding an RFID source [44], with the vertical axis representing voltage and the horizontal axis representing time.

### 4.3. Proof-of-Concept: AlexNet on CIFAR10

**Dataset:** We use CIFAR10 for RGB images of size 32 × 32. It has 60,000 images evenly distributed in ten distinct classes, of which 50,000 and 10,000 examples are used for training and testing, respectively. CIFAR10 has gained widespread recognition as a widely used benchmark dataset in the domains of machine learning and computer vision for image classification tasks. The selection of this dataset was driven by its well-established nature and the extensive usage it has received in the field.

**Neural network architecture:** We specifically chose the AlexNet neural network architecture, renowned for its successful utilization in various image analysis tasks. The AlexNet architecture is known for incorporating multiple convolutional layers, which have proven to be highly effective in extracting meaningful features from images. It is important to note that the original design of the AlexNet architecture was primarily tailored for larger input sizes, typically 227×227 images. Consequently, there may be concerns regarding its applicability and effectiveness when applied to datasets with smaller image dimensions, such as CIFAR10. However, we addressed this potential limitation by meticulously adjusting the hyperparameters of each layer to adapt the model and optimize its performance specifically for accommodating the CIFAR10 database [45]. This approach enabled us to overcome the inherent challenges associated with the discrepancy in image dimensions and attain a high level of effectiveness in our experiments.

### 4.4. Performance Evaluation

**Setup:** To accurately assess the power consumption, we utilized the gem5-Aladdin simulator as a crucial tool in our evaluation process [46]. The gem5-Aladdin simulator offers a comprehensive framework for computer architecture simulation by combining the functionalities of gem5 and Aladdin, two widely used simulation tools. To capture the specific power consumption characteristics of our designs, we made specific modifications to the configuration of the gem5-Aladdin simulator. To ensure an accurate representation of power consumption, we extended the capabilities of the gem5-Aladdin simulator to account for the power consumed during read-and-write operations performed on non-volatile registers. By modifying these operations, we incorporated the power consumption associated with these operations into our evaluation. The gem5-Aladdin simulator mainly operates on C code for computation. However, due to limitations in analyzing two-dimensional (2D) arrays within the gem5-Aladdin framework, we opted to convert these arrays into one-dimensional (1D) arrays. This conversion allowed us to effectively analyze and evaluate the power consumption of our proposed architectures without compromising the accuracy of the results. To estimate energy consumption and latency, based on Table 1, and to calculate power, we divided the energy by time. This table provided valuable information regarding the energy and latency values associated with both read and write operations. Based on this table, we ensured that our power and latency estimations were based on relevant and reliable data, contributing to the robustness of our evaluation process. Furthermore, as mentioned above, we considered $tr_{1}$ = 2.5 V, $tr_{2}$ = 1.5 V, and $tr_{3}$ = 0.8 V.

**Figure 11:**
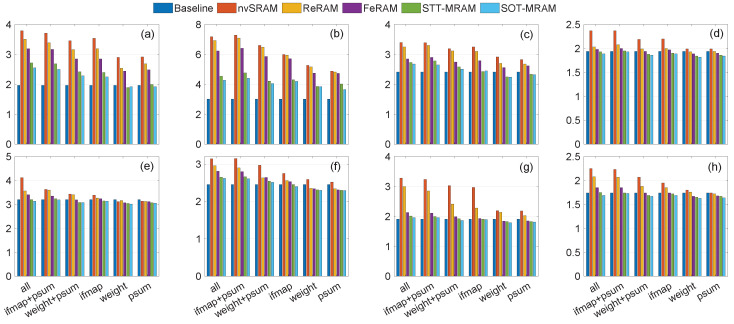
Power consumption (mW) of different scenarios using the RS dataflow, as a result of various eight various traces (**a**–**h**) shown in Figure 10.

**Figure 12:**
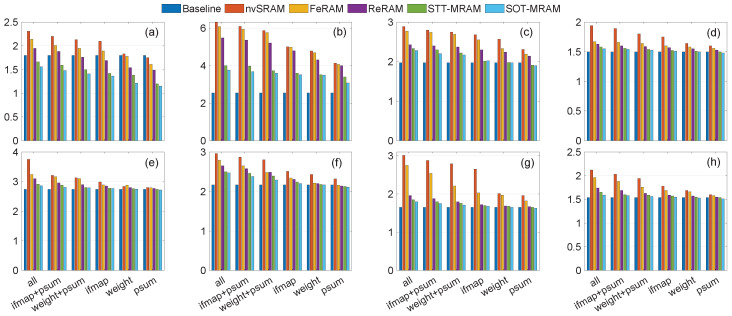
Power consumption (mW) of different scenarios using the WS dataflow, as a result of various eight various traces (**a**–**h**) shown in Figure 10.

**Figure 13:**
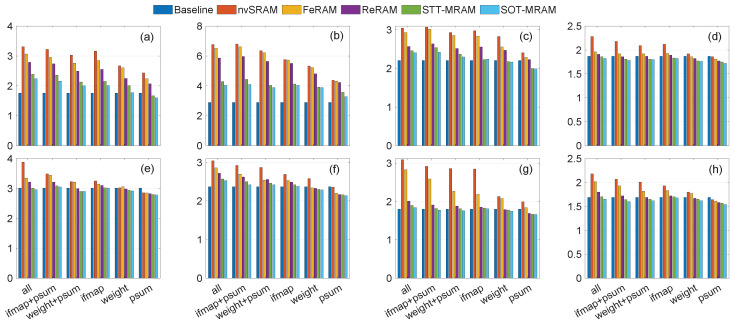
Power consumption (mW) of different scenarios using the OS dataflow, as a result of various eight various traces (**a**–**h**) shown in Figure 10.

The experimental results presented in Figure 11, Figure 12 and Figure 13 demonstrate instances where lower power consumption is observed, which can be attributed to reduced computational requirements stemming from the preservation of data in non-volatile registers. Moreover, eliminating transmission between the PE and off-chip DRAM contributes to the overall reduction in power consumption. As shown in Figure 11, Figure 12 and Figure 13, when the non-volatile backup is enabled for all registers, power consumption increases in all traces. As a result of reducing the number of non-volatile registers, power consumption decreases. In some traces, such as trace Figure 10b, the power consumption is higher even when using only one non-volatile register to store a single integer, compared to the baseline where no non-volatile registers are used to save data during power failures. This scenario represents the worst-case for our trace as the voltage level frequently drops below $tr_{2}$. Consequently, it necessitates backup from all registers while ensuring that the voltage never falls below $tr_{3}$. As a result, the system remains continuously powered on, eliminating the need to restore the value from the NVMs. However, in other traces, we observed that in certain scenarios (particularly when saving only the ofmap, which requires saving one integer, or the weight, which requires saving three integers), the power consumption is lower than the baseline. This suggests that storing data in NVMs can be more power-efficient compared to the power of transferring data from the main memory to each PE and recalculating the psum from scratch. Additionally, if we scale up our structure, targeting larger spatial dimensions for filters/inputs, and CNN architecture, this power-saving feature becomes more pronounced as the number of memory references significantly increases. By storing data in NVM, the power consumption associated with data transmission and recomputing from scratch can be eliminated, enabling instant computing. Furthermore, by using new technologies that have lower read and write power consumption, such as SOT-MRAM, in some scenarios, we have less power consumption than the baseline. However, using additional NVMs imposes memory area overhead ranging from 16% to 100%, where psums’ registers and all PEs’ registers include extra non-volatile elements, respectively.

**Figure 14:**
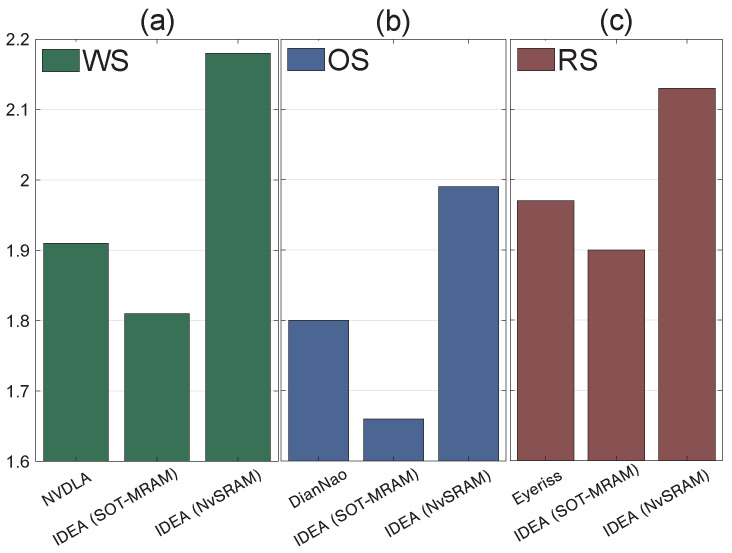
Power consumption (mW) comparison for different studies using various traces; (**a**) [31] for trace 4, (**b**) [37] for trace 7, and (**c**) [25] for trace 3.

Figure 14 presents a comparative analysis of different implementations within our design framework. We explored two distinct scenarios by integrating non-volatile technologies, specifically SOT-MRAM and NvSRAM, and benchmarked them against three existing implementations: Eyeriss [25], NVDLA [31], and DianNao [37]. This comparison aims to highlight the performance and efficiency differences between our proposed solutions and established designs in the field.

In addition to slight improvements in power consumption using IDEA, the obtained results indicate the number of cycles required to perform AlexNet’s operations are summarized in Table 2. On average, we observed an enhancement in the number of cycles by approximately 21%. This reduction in computational cycles can be interpreted as lower energy consumption. The energy consumption of DRAM for input activation is notably high. Integrating NVM within PEs can present a more energy-saving alternative than the conventional approach, where all registers are volatile. With volatile registers, any power interruption requires the system to reboot, which mandates fetching the ifmaps and weights from DRAM again. This introduces latency into the system’s recovery process and entails additional energy consumption, as accessing and retrieving data from DRAM is a relatively power-intensive task. As an illustration, our tests using AlexNet on the CIFAR10 dataset indicated a 2.7% enhancement in power efficiency with the proposed architecture, as shown in Figure 14.

## 5. Discussion and Future Work

### 5.1. Observation 1

Our results demonstrate that the combination of the RS dataflow and SOT-MRAM technology yields the best performance and power efficiency improvements. The RS dataflow is designed to optimize data locality and minimize data movement, which is crucial for reducing energy consumption in computational processes. By keeping data operands stationary within processing elements as much as possible, the RS dataflow maximizes data reuse and minimizes the energy-intensive operations of data transfer between memory and processing units. This approach not only leverages spatial locality by processing data points in close physical proximity, but also significantly reduces the energy costs associated with data movement.

The traditional architectures often incur substantial power consumption due to frequent data transfers between memory and processing units, which represents one of the largest components of overall energy expenditure in computational systems. This inefficiency is further exacerbated in high-performance computing applications, where the volume and velocity of data movement can dramatically inflate energy costs. In addressing this critical challenge, our approach significantly curtails the power consumed in data movement, which is traditionally the most energy-intensive aspect of system operation. By optimizing data locality and minimizing unnecessary data transfers, the RS dataflow inherently reduces the bulk of power consumption that characterizes conventional computational processes. SOT-MRAM technology complements the RS dataflow with its fast switching speeds, low power consumption, and high endurance. The integration of SOT-MRAM into our architecture enhances the energy efficiency and performance of the system by providing a reliable, high-speed memory solution that aligns with the RS dataflow’s requirements for minimized energy expenditure and maximized computational throughput. The low energy consumption and durable nature of SOT-MRAM, coupled with its ability to preserve data integrity even under intermittent power conditions, ensure that computations can proceed efficiently without data loss. Given that data movement accounts for a considerable portion of the system’s total power consumption, our innovative combination of RS dataflow and SOT-MRAM technology directly target and mitigates this primary energy drain. This strategic alignment not only enhances overall system efficiency but also represents a pivotal shift towards more sustainable computing architectures, where the reduction of energy spent on data movement is paramount. These enhancements are critical in the context of modern computing, where power efficiency is not merely an operational advantage but a fundamental requirement for scalability and environmental sustainability. By significantly diminishing the power consumed through data movement—traditionally the dominant factor in a system’s energy profile—our architecture sets a new benchmark for power-efficient computing.

In the combination of RS and SOT-MRAM scenario, we achieved a 2.7% gain in power efficiency and a 21% reduction in required cycles. This substantial improvement in both power efficiency and computational speed underscores the effectiveness of our approach, demonstrating that the combination of RS dataflow and SOT-MRAM technology is particularly effective, yielding the best-observed improvements in performance and power efficiency by effectively reducing energy consumption while enhancing system reliability and speed.

### 5.2. Observation 2

As the landscape of memory technologies rapidly evolves, our study delivers critical insights aimed at refining systolic array designs, marking a pivotal step toward the next generation of hardware enhancements. These enhancements are instrumental in reducing energy consumption and boosting the speed and efficiency of forthcoming computing systems. By integrating computing technologies capable of handling interruptions with non-volatile memory, the proposed IDEA architecture sets a robust groundwork for advancements in eco-friendly computing. The relevance of our findings is poised to escalate as energy harvesting gains traction, offering invaluable strategies for developing systems optimized for low-power scenarios, thereby contributing significantly to the field of sustainable computing.

### 5.3. Future Work

The significance of our work lies in its contribution to the ongoing effort to optimize computing systems for energy conservation and operational efficiency, addressing the critical challenges faced by the IoT ecosystem. Looking forward, we envision several promising directions for future research. Exploring the application of our findings to a broader array of workloads and computing environments could further elucidate the versatility and scalability of the IDEA architecture. Additionally, investigating the integration of emerging NVM technologies that offer even greater energy savings and performance benefits holds the potential to substantially advance the state of the art. Finally, the development of adaptive dataflow strategies, leveraging advances in machine learning and artificial intelligence, could enable dynamic optimization of computing resources in response to varying workloads and operational conditions. By pursuing these avenues, future research can build on our work to unlock new possibilities in the design of efficient and effective computing systems for the IoT and beyond.

## 6. Conclusions

This paper delved into the evolving realm of intermittent computing within energy-constrained IoT environments, highlighting a comprehensive study that spans various dataflows and NVM technologies. Our research, transcending the confines of a singular architectural model, demonstrated significant performance and energy efficiency enhancements across a spectrum of systolic array designs. Notably, in our empirical evaluation with AlexNet on the CIFAR10 dataset for the proposed IDEA architecture, we achieved a 2.7% gain in power efficiency and a 21% reduction in required cycles in the best-case scenario. These results illustrate the profound impact of integrating diverse NVM technologies with different dataflow methods. Through this integration, we pave the way for next-generation computing systems that are more energy-efficient, robust, and capable of operating effectively in energy-constrained environments. The adoption of such advanced technologies and methodologies signifies a critical step towards the realization of sustainable computing ecosystems capable of supporting the increasing demands of the IoT landscape. Our findings lay a foundation for the continuous evolution of computing architectures, promising significant strides in performance, power efficiency, and environmental sustainability 

## Figures and Tables

**Table 1:** Features of non-volatile technologies.

Technology	nvSRAM [21]	ReRAM [22]	FeRAM [23]	STT-MRAM [24]	SOT-MRAM [24]
Read time (ns)	0.22	5	60	5	10
Write time (ns)	0.24	5	60	10	1
Read energy (fJ)	50	2700	9000	10	10
Write energy (fJ)	3000	2700	9000	100	10
Read Power (mW)	0.22	0.54	0.15	0.002	0.001
Write Power (mW)	12.5	0.54	0.15	0.01	0.01

**Table 2:** Number of required cycles performing the AlexNet considering the target traces in Figure 10.

**Trace in Figure 10**	**Baseline**	NVM-Enhanced IDEA			Improvement (%)		
		RS	OS	WS	RS	OS	WS
(a)	2276	1748	1935	2048	23.16	14.9	10
(b)	7813	7715	7731	7780	1.25	1.04	0.42
(c)	5117	3525	4349	4505	31.10	15	11.9
(d)	3564	2930	3046	3194	17.80	14.6	10.38
(e)	8038	6010	6398	6787	25.19	20.4	15.56
(f)	6561	5103	5477	5801	22.22	16.52	11.58
(g)	4019	3364	3571	3784	16.3	11.14	5.84
(h)	3753	2943	3103	3309	21.58	17.37	11.83

## Data Availability

Data are contained within the article.

## Abbreviations

The following abbreviations are used in this manuscript:

NVM	Non-volatile memory
PE	Processing Element
ASIC	Application-specific integrated circuit
CNN	Convolutional Neural Networks
MAC	Multiplication-and-Accumulation
ifmap	Input Feature Map
ofmap	Output Feature Map
W	Weight
WS	Weight Stationary
OS	Output Stationary
RS	Row Stationary
GLB	Global Buffer
CHRT	Cascaded Hierarchical Remanence Timekeepe
ReRAM	Resistive Random Access Memory
FeRAM	Ferroelectric RAM
nvSRAM	Non-Volatile Random Access Memory
SOT-MRAM	Spin-Orbit Torque Magnetic Random Access Memory
STT-MRAM	Spin-Transfer Torque Magnetic Random Access Memory
